# “Our services are not the same”: the impact of the COVID-19 pandemic on care interactions in women’s shelters

**DOI:** 10.1186/s12905-023-02541-7

**Published:** 2023-08-11

**Authors:** Caitlin Burd, Isobel McLean, Jennifer C. D. MacGregor, Tara Mantler, Jill Veenendaal, C. Nadine Wathen

**Affiliations:** 1 Faculty of Information and Media Studies, Western University, 1151 Richmond St, N6A 5B7 London, ON Canada; 2 School of Architecture and Landscape Architecture, University of British Columbia, Vancouver, BC Canada; 3 Arthur Labatt Family School of Nursing, Western University, London, ON Canada; 4 School of Health Studies, Western University, London, ON Canada; 5 Mobilizing Knowledge on Gender-Based Violence, Arthur Labatt Family School of Nursing, Western University, London, ON Canada

**Keywords:** COVID-19 pandemic, Women’s shelters, Gender-based violence, Intimate partner violence, Service delivery, Public health

## Abstract

**Background:**

Guidelines and regulations in response to the COVID-19 pandemic have significantly impacted the health care sector. We explore these impacts in the gender-based violence (GBV) services sector and, more specifically, in the context of women’s shelters.

**Methods:**

Using an interpretive description and integrated knowledge mobilization approach, we interviewed 8 women’s shelter clients, 26 staff, and conducted focus groups with 24 Executive Directors.

**Results:**

We found that pandemic responses challenged longstanding values that guide work in women’s shelters, specifically feminist and anti-oppressive practices. Physical distancing, masking, and closure of communal spaces intended to slow or stop the spread of the novel coronavirus created barriers to the provision of care, made it difficult to maintain or create positive connections with and among women and children, and re-traumatized some women and children. Despite these challenges, staff and leaders were creative in their attempts to provide quality care, though these efforts, including workarounds, were not without their own challenges.

**Conclusions:**

This research highlights the need to tailor crisis response to sector-specific realities that support service values and standards of care.

## Background

The COVID-19 pandemic has severely impacted everyday life across all social contexts and work sectors, especially the health and social service sector. However, vulnerable groups, including women experiencing violence, have been disproportionately affected [1]. Gender-based violence (GBV) is a major public health issue and human rights violation with estimates that 65% of women are exposed, either directly or indirectly, to at least one form of GBV in their lifetime [2]. Evidence demonstrates that survivors suffer a range of negative health impacts as a result of experiencing violence [3, 4]. Experiences of GBV, in both frequency and severity, increased during the pandemic [2, 5–7] with pandemic protocols, such as stay-at-home orders, increasing risks for women at home [8–11]. Thus, for many women and children experiencing violence, home was not a safe place to be. Many agencies and crisis lines reported a higher need for services for women and children as a result of the pandemic; however, pandemic protocols limited the ability of GBV agencies to provide what felt like adequate and/or sufficient services [7, 10–13]. Historically, GBV services, and specifically women’s shelters, have faced the difficult task of trying to stretch limited resources within a fragmented social service system to provide quality care to clients and meet their needs [14, 15] the pandemic exacerbated these challenges and changed the way the work could be done [2, 7, 9, 11].

Women’s shelters are one crucial part of system services instituted to support the health and wellbeing of women and children experiencing violence, and serve as a key referral point to and from other services, including health services [14, 16, 17]. Though the literature is sparse on the topic of women’s shelter spaces and care interactions, it is known that communal spaces (e.g., kitchens, living rooms) are important for the development of a sense of safety and community [18]. Guidelines for shelter design based on a comprehensive literature review of women’s experiences in shelters across North America also listed ‘Creating a Sense of Community’ [19, 20] as one of the key aspects of designing supportive spaces for GBV survivors. However, research has demonstrated the negative impact of the pandemic on fostering a sense of community through communal spaces; pandemic restrictions reduced available communal spaces in shelters by 48% [21] and sparked safety concerns with the use of alternative spaces, like motels/hotels, as well as concerns about how quality care and empowerment can be maintained under pandemic conditions [22, 23].

Research has outlined the importance of relationship building and connection in women’s shelters, such as the importance of ‘caring citizenship’ [24, 25], the dialogue between givers and receivers of care, which contributes to an environment of quality social care [25]. Research from care-focused professions, such as counseling, has shown that therapeutic relationships were negatively impacted during the pandemic by mask mandates that impeded the exchange of visual cues between counsellors and clients [26]. Furthermore, in the GBV sector, research has demonstrated that isolation requirements for those accessing services felt restrictive and controlling, and, for some survivors, replicated core aspects of the abusive relationship from which they were seeking refuge [27, 28]. These findings highlight conflict between ‘one size fits all’ pandemic protocols and the core values of most GBV agencies, such as trauma- and violence-informed, feminist and anti-oppressive care, all of which center reducing the risk of further harm through understanding the impacts of trauma, prioritizing all forms of safety, and promoting capacity-building, collaboration, and choice [29]. This conflict between the realities of service provision during the pandemic and the core values of GBV services runs the risk of re-traumatizing women and their children [7, 15, 28].

Some research has explored the use of alternative modes of service delivery to adhere to pandemic protocols and how this impacts the quality of service. For example, research on the use of alternative spaces by GBV agencies showed that connections between clients and staff were significantly hindered, in part because it was difficult to get in touch with clients or effectively work on transitional housing plans together [11, 22]. A few studies in the United States have explored the use of virtual services as alternatives to in-person service (e.g., emailing with clients) and the impact on service accessibility, client satisfaction, and staff perceptions. This research has demonstrated that the rapid shift to remote or virtual services was useful in ensuring clients continued receiving support [30] and tended to be received positively by clients [15, 31]. However, there were challenges with online service models, such as safety risks, device security, the creation of extra work for counsellors [30], access to technology [9, 15], and difficulties for rapport-building and emotional connection, particularly for clients with disabilities or those who did not speak English [15, 31]. Similar Canadian findings showed that virtual or remote services were perceived as the best option to continue to support clients but presented challenges for building rapport and maintaining connection [32]. A Canadian survey of women accessing GBV outreach services found that some women experienced remote service delivery methods (video, phone, text) as more accessible, while others hoped to return to in-person care [9]. Complicating the move to virtual or other creative service formats, some research suggests that during the pandemic clients were presenting with more complex cases and compounding issues (e.g., mental health, addictions, severe trauma) that were not easily supported by these new service models. Additionally, services often suffered when clients struggled with equipment, technology (e.g., stable internet connection), and technological skills required for virtual care [13, 15].

Most research to date on the impact of the pandemic and associated guidelines on service delivery in the GBV sector has focused on new and innovative ways to connect with clients when pandemic protocols limited in-person services. While some research has described the impacts for women of isolation requirements and restrictions on space in GBV residential care, there has been little attention on how other requirements, such as masking, physical distancing, and restrictions on communal spaces, affected service provision for women in shelters. Given this gap in the literature, the current paper aims to provide further understanding on how COVID-19 pandemic protocols impacted care interactions within women’s shelters in Ontario, Canada.

## Methods

### Overview

The current analysis is drawn from a larger, qualitative research project that analyzed the impact of the COVID-19 pandemic on various aspects of the GBV sector in Ontario, Canada. This analysis uses data collected from GBV service staff, Executive Directors (EDs), and women using services. We used interpretive description (ID) methodology alongside an integrated knowledge mobilization (KMb) approach. ID is a qualitative methodology that prioritizes the centering of multiple perspectives and acknowledges the co-construction of knowledge between researcher and participant [33, 34], with a focus on practice-based, action-oriented knowledge generation, which is an excellent fit with an integrated KMb approach [35, 36]. We co-designed the research with leaders from five GBV service organizations who were formal study partners and ensured the creation of mutually beneficial research and recommendations for action. This framework allowed for a reflective and iterative process for data collection and analysis. Ethics approval was obtained from Western University’s Non-Medical Research Ethics Board (Protocol 115,865). All methods were carried out in accordance with relevant guidelines and regulations. Additional information on our methodological approach is available [22].

### Sampling and procedures

Participants were recruited through purposive and snowball sampling; emails were sent by sectoral list-servs and partnering EDs shared the study with relevant parties, such as GBV agency staff. Informed verbal consent was obtained from each interested participant who met the inclusion criteria. All staff and women who participated in this study were offered a $50 gift card in recognition of their time, while EDs were not offered compensation.

GBV staff (*n = 26*) and women using GBV services (*n = 8*) were interviewed individually by telephone or video chat; interviews lasted up to 60 min and took place between June and October 2020. Focus groups with 24 EDs of GBV or related agencies were facilitated by two research team members over Zoom; these five focus groups occurred between June and October 2020 and each lasted up to two hours. All interviews and focus groups were audio-recorded, professionally transcribed verbatim, anonymized, and checked for errors before analysis.

### Participants

Participants included in this research either led, provided, or accessed services within a GBV or related agency, representing 24 different agencies across Ontario. Staff and EDs served a variety of community sizes, ranging from 4,700 to 1,500,000, with ten EDs at agencies in a rural setting, two EDs identifying their agency as serving primarily Indigenous clients, and 58% at abused women’s shelters. The majority (65%) of staff were employed full-time, 15% identified as relief or casual, and 25% worked remotely during the pandemic. The work roles of staff varied, with the following main areas of work: residential counselling (54%), sexual assault services (15%), outreach (8%), and support (e.g., custodial, food preparation; 4%). The majority (83%) of EDs were female-identified, while all staff and all clients identified as women. Participants accessing services were all female-identified, about half of those accessing shelter space had never used shelters before, and 63% had children with them in the shelter, most of whom were under 2 years old. A complete description of participant characteristics is available [28].

### Data Analysis

Interview and focus group transcripts were organized and coded using Quirkos [37] qualitative analysis software. A preliminary codebook was developed by members of the research team to guide analysis. The codebook evolved through an iterative process, as each transcript was coded independently by two research team members. Next, each pair discussed the codebook and the coding process, and subsequent meetings were held with all coders to discuss potential revisions to the codebook and wider themes present in the data. Once all transcripts were coded, they were merged in Quirkos, resulting in three data files (women’s interviews, staff interviews, ED focus groups) capturing the coding of all team members. As themes were identified specific to care interactions, research team members conducted queries in Quirkos, key word searches (e.g., “care”, “caregiving”, “emotional labour”), and closer analysis of specific quotes coded under relevant themes. Preliminary findings were discussed with our GBV service partners at a two-day knowledge sharing event to ensure that themes were reflective of first-hand experiences in the field, as aligned with interpretive description [34]. The findings below represent an array of participant voices and multiple (including converging and diverging) perspectives.

## Results

We have managed to do what we can within the parameters of what we are dealing with [during the pandemic], but our services are not the same… We are managing, we are adapting, we’re showing incredible resilience, but… people should not pass a pandemic [in] a shelter. It’s not the place. (Focus Group [FG] 205)

**Fig. 1:**
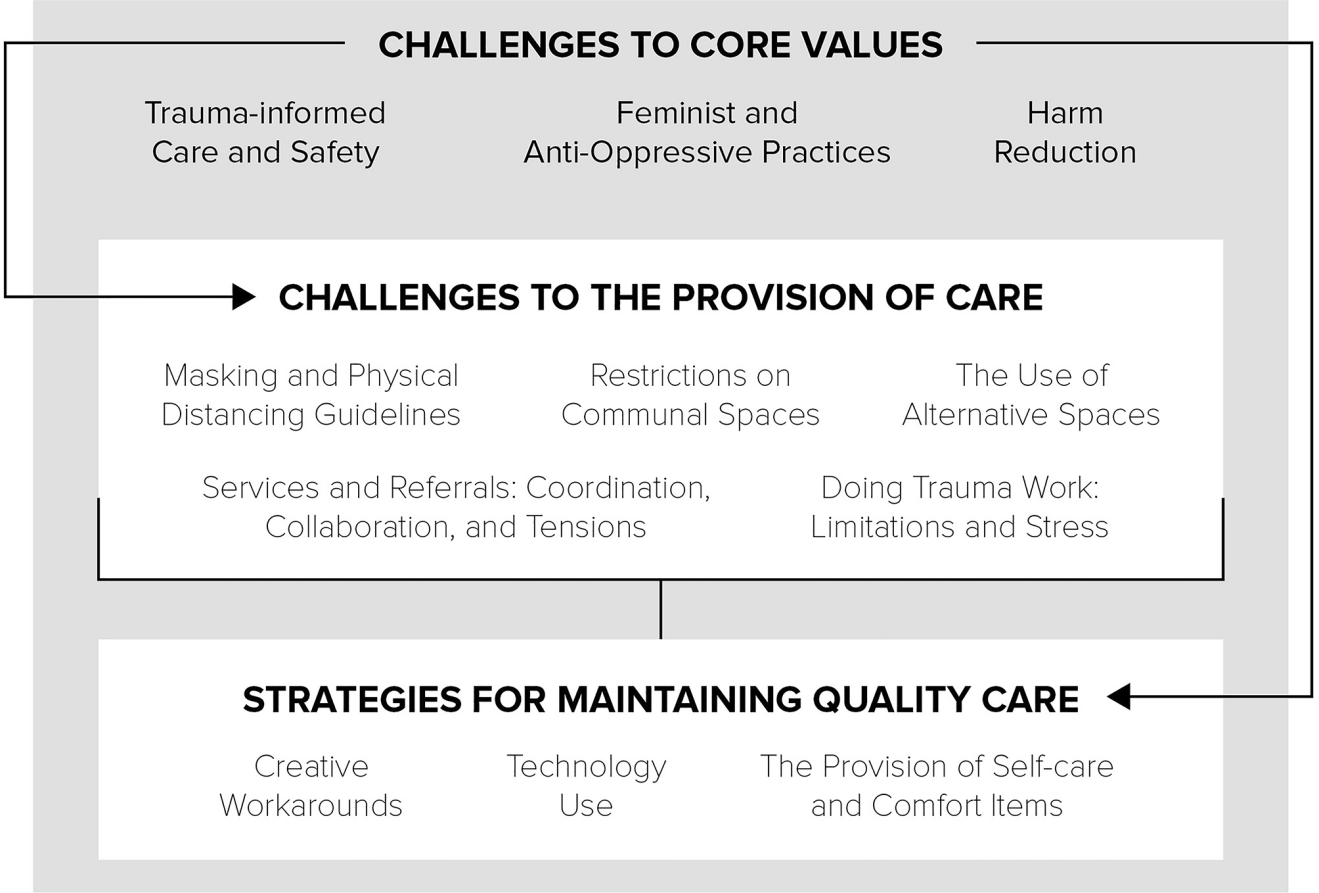
Interconnections of Main Themes

Work within women’s shelters is often complex, and the COVID-19 pandemic interrupted care in these agencies in multiple ways. The findings are organized into three interconnected themes, each with various sub-themes (see Fig. 1): (1) challenges to core values (i.e., maintaining principles at the heart of the work), (2) challenges to the provision of care (i.e., how pandemic protocols impacted what could be done), and (3) strategies for maintaining quality care (i.e., workarounds that staff employed to maintain or reclaim quality care interactions).

### Challenges to core values

Central to work in the GBV sector and, more specifically, women’s shelters, are values that guide service provision, especially feminist and anti-oppressive, trauma-informed and culturally safe practices, and, for some services, harm reduction. During the COVID-19 pandemic, guidelines often contradicted or challenged these core values: “… [our values have] all been greatly compromised during this pandemic” (FG202).

#### Feminist and Anti-oppressive Principles

The pandemic challenged feminist and anti-oppressive practices underpinning service interactions with clients; agencies had to consider how changes to their service models (e.g., virtual services, the closures of some services, motels, etc.) that were necessary to address pandemic conditions would also impact women and children using services. One ED (FG201) discussed how the feminist principle of choice could be upheld and how changes to services impeded their ability to meet women where they were at,

How do we engage with women in designing what she wants her service with us to look like? What does she hope will change? What’s broader here? And so, [the pandemic] ended up having pretty deep [impacts] in changing our language, changing… how we position the range and scope of our services.

Women also felt that practices in shelter during the pandemic strayed from feminist principles of centering women’s experiences and working to reduce oppression, as some felt that their voices took a back seat to pandemic protocols, as one woman (Woman [W] 128) explained,

It was like [some staff] didn’t even look at you like a person, kind of, you were like a number almost… There was a lot of ‘if this doesn’t happen, we can get shut down,’ so I think that there were certain staff who looked at it like ‘well, if you don’t do this, you’re not going to have a place to go’ kind of thing.

Some shelters altered their scope of service through mandate changes to ensure that those at the highest risk of violence were served. However, this reduced the number of women and children who could receive support and those who did not meet certain criteria were left with few service options, including stays at local motels/hotels. Some women also experienced intense questioning about their abuse experiences (i.e., as to whether their circumstances fit new mandates) when they were trying to access shelter space, which made them feel like staff or the agency did not care about them. For example, one woman (W128) said, “… to get a spot in here… they made me jump through hoops and scramble and struggle and fight… I felt like a piece of garbage on the street.” This woman had not seen her abuser in three months and, as such, felt the shelter did not take her experiences seriously — that there was not enough risk to warrant giving her one of the few shelter beds available. At the same time, in the wake of public health restrictions that limited available space and restricted services, staff struggled with having to turn women and their children away with fewer options for referrals, which made some staff and women feel that adequate GBV services were unavailable during the pandemic. One staff member (Staff [S] 124) said, “… there [have] been some hard conversations around, you know, I had to say no to a woman who just wasn’t high risk enough and now she doesn’t have somewhere safe to stay. So, it’s been hard.”

There were also challenges related to promoting women’s empowerment, as attempts by shelter staff to foster independence and healing were significantly hindered by restrictive pandemic guidelines. These guidelines had women and children leaning more on staff for basic needs (e.g., food items) and, in some cases, staff were required to accompany women on outings. One ED (FG205) shared her perspective that independence and decision making within shelters were stripped from women, “And this weird sense that we have to manage women’s decision making for [them] and their families’ health… I think it’s… looking for the ways to operationalize the feminist values in our responses to COVID.” Staff also felt that new pandemic guidelines were sometimes oppressive; they felt they were policing women and children by having to closely monitor their actions to adhere to the protocols. One staff member (S121) said,

You feel like you’re babysitting, and just from like a feminist perspective it’s so not empowering to a woman at all to be like, ‘OK, well I’ll come with you to do this and do that’ … it just felt so oppressive.

#### Trauma-informed approaches

Many of our staff and ED participants aligned their work and/or their agencies with trauma-informed care, which emphasizes the importance of understanding, and taking into account, the impacts of trauma and especially in this case, intimate partner/domestic violence. Unfortunately, pandemic guidelines, such as isolation requirements, restrictions on access to communal spaces, and curfews, sparked re-traumatization for women who had similar experiences with their abusers. As one woman (W122) shared,

[Isolation] was a little difficult, coming from a traumatizing experience, because I was very isolated in the relationship that I had fled from. So, it kind of brought up a lot of recurring anxieties, to be secluded and isolated and not be allowed out.

Staff and EDs felt these requirements were in conflict with their values, with one ED (FG205) saying, “Isolation is counterintuitive to the work that we do. Our entire purpose and job, besides safety and options and all, is to break the isolation… All of a sudden, isolation is the defining moment of 2020.” There were also views that strict masking mandates were not trauma-informed and did not allow flexibility for women or children who were experiencing trauma and uncertainty in their lives. For example, one staff member (S120) said,

Some of the recommendations that they’re making about mandatory masking, you know, again, from a trauma- and violence-informed perspective, that can really impact somebody’s care. So, we take the steps and measures to be able to safely engage them through the use of other personal protective equipment [PPE], barriers and whatnot, so that way, folks aren’t having to wear something that’s going to impact their ability to engage in program or feel safe within the scope of the services that we offer.

Linked to trauma-informed care, the value of safety and how to achieve it took on a somewhat different meaning during the pandemic. The COVID-19 pandemic extended the issue of safety from a focus on abuse and trauma to a focus on physical safety from the virus and PPE use, the potential use of the virus and protocols as forms of abuse by the perpetrator, and the impact of pandemic protocols on safe service provision, as one ED (FG205) said,

Safety has taken a new meaning through COVID, now it’s physical safety more so than the other things that we normally worry about… PPE. Masks. Six feet apart. Hand sanitizer… it’s all that kind of stuff that is first and foremost on people’s minds.

Safety from the virus had to be prioritized over other important values, like community partnerships and collaboration or trauma-informed care. Some staff and EDs reported that public health and funding ministries were prioritizing the enforcement of pandemic protocols (such as using PPE), rather than the quality of care:

One of the first conversations we had with public health, who have been amazing and wonderful, was about how when new clients would come into shelter we would have to ask them to quarantine in their room for 14 days. And then, having conversations with public health about how that is incredibly triggering for women who have experienced violence, especially if they have been confined in their homes by their abusers and how difficult this is going to be. (FG204)

#### Harm reduction

Not all agencies in our study were grounded in harm reduction, which, in contrast to abstinence-based approaches, is a philosophy, and set of related strategies, to reduce or mitigate the negative impacts of high-risk behaviours, such as sex work and substance use. While most of the agencies represented by the EDs participating in our focus groups took a harm reduction stance, this was not the case among the staff interviewed, who represented many different agencies, and organizational policies, on substance use and mental health. In many cases, adjustments had to be made for several aspects of shelter work. For example, some agencies implemented changes to their substance use policies such that staff purchased alcohol or helped procure cannabis (which is legal in Canada) to align with public health guidelines that encouraged limitations on community outings: “We were having to go out and purchase their things that they needed for harm reduction, allowing them to use on-site when we don’t allow that usually…” (S125).

For some staff and EDs, adjusting or adopting harm reduction policies and practices improved support for women who were actively using during their shelter stays. The change also improved communication and transparency between staff and women, allowing for more supportive care to be provided when it came to managing substance use. However, other staff felt conflicted about supporting women’s substance use because it was inconsistent with their own personal values. Additionally, some staff felt that new harm reduction processes, amidst other changes due to the pandemic, were quite harmful: “What I worry about is that we’ve created such a codependent relationship with residents… they’re not doing any of the cooking on their own… they come to us for their marijuana…” (S115).

Discussions about harm reduction also extended to balancing the needs of women and their children with new pandemic protocols or guidelines and trying to minimize the potential for unintended harms. One ED (FG205) shared how the value of harm reduction broadened in scope during the pandemic, “… a harm reduction approach, I think, was helpful [during the pandemic] in making decisions [about] how to keep people safe in the shelter…” Furthermore, EDs discussed the prioritization of certain pandemic-required values as an effort to minimize harm, as best they could, while balancing their longstanding values; one ED (FG205) said,

It’s easy to get distracted by the fact that we’re dealing with a pandemic. So how do we also hold those critically important pieces to live up to our values even though [we have] shifted over to just figure out how much more PPE and everything we need.

### Challenges to the Provision of Care

The provision of care and what we have defined as ‘trauma work’ (i.e., counselling and supporting women and children who have experienced abuse and trauma) is an important but sometimes informal and invisible aspect of women’s shelters. Much of this work is done by counsellors in shelters, but also in a less formal capacity by other shelter staff who have contact with women, such as cooks or custodians. Informal and formal connections are incredibly important for fostering a sense of community, support, and healing within residential services but they were severely limited by pandemic guidelines such as masking, physical distancing, the use of hotel spaces, and closures of communal and counselling spaces. Staff shared that pandemic protocols and the pandemic in general detracted from valuable work with clients. For example, one staff member (S116) said,

And right now, it’s really hard for us to give [clients] all the time and attention that they truly need to manage their addictions and manage their mental health… We’re doing the best we can, it’s just really hard because we have so many things to do constantly all day now with COVID.

#### Masking and physical distancing

Several COVID-19 precautions made providing service and care to clients more difficult and forced staff to focus more on policing women and children and keeping up with ever-changing guidelines, rather than directly supporting clients. Two of the most prominent that our participants identified as impacting care interactions were masking and physical distancing.

Most agencies in our study required all staff and women to wear masks within the shelter, a common practice across most work sectors in Ontario during the pandemic. However, in a setting with traumatized clients with violence experiences, masking protocols hampered the ability of staff to read visual/non-verbal cues and connect on an emotional level, as one staff member (S124) said, “… it just makes it hard when you’re meeting with another person and maybe they’re in crisis and you’re both wearing a mask. Just the relational piece can be difficult…” Staff also identified that trauma impacted some women’s ability to comply with masking guidelines: “And then there’s the other clients who, because of their trauma, can’t wear a facemask and then they’re already coming through the door with so much shame… [it] just adds another – a whole other layer of shame to that” (S112). Staff were able to use alternative PPE, like face shields or plastic barriers, to connect with women who could not mask or in instances where language was a barrier, as one staff member (S120) indicated,

We are mandated to have them masking, and, again, we use that with the discretion that if it’s going to impact the care that we’re offering to women or able to serve a woman, then we are not going to mandate [the] mask but our staff will take those steps and measures to ensure that there is distancing, our staff can wear a mask. We have additional PPE to put in place. But, again, the masking piece was just - it was just problematic and impacted the care that we could offer to some folks.

As well, in some cases, staff put their own physical health and safety at risk to prioritize connecting with clients that they were meeting in person: “And the tension it creates for some of our staff who refused, frankly, to wear PPE in the beginning because they valued making that connection with their client over their own safety” (FG203). However, these workarounds still left staff feeling that care was impacted and their ability to connect with women and their children was greatly hindered.

Physical distancing requirements often prevented staff from meeting face-to-face with clients, something that is crucial in a shelter context. Staff connected with women using email or phone calls, diminishing their ability to develop rapport and help women through their trauma:

The work, it just isn’t the same. There’s nothing like sitting in a room with someone and they get it, or you know that you’re truly being heard versus on the phone with somebody. I have no idea what they look like or what their reactions are to what you’re saying. It’s just … less personal… (S102)

Staff felt that not seeing their clients face-to-face affected their ability to assess mental health or other needs. In contrast, a few staff who had smaller caseloads (due to distancing guidelines that reduced available beds in shelter) and fewer clients on-site in the shelter, reported that they were better able to manage their caseload and give more time to their clients to discuss their trauma and goals. For example, one staff (S105) said,

Because if I had, like, seven women on my caseload, I can’t spend 30 minutes of my day talking to one of them about their past trauma… I want to, but I can’t. But now [during the pandemic], I can, and both of the women on my caseload very much appreciate that. And, they really need it.

The ability to provide emotional support was also negatively impacted by distancing guidelines for women: “… if somebody is crying, or upset, normally I would ask [them] if they wanted a hug or something. But I can’t do that anymore… I understand the COVID guidelines… but it’s just, it feels so insincere…” (S110), and children:

… we had a lot of young children who really didn’t understand [the guidelines]. We’re not allowed to be hands-on with them because of COVID, so, when they run up and try to hug me or ask me to carry them, I have to say no. (S118)

And then, the kids socializing in the shelter was half the reason it made it easier for them to come there, because they made new friends. And that didn’t happen, right? Like, yesterday, I had a little baby that was new, and she came in and she wanted to play with the other baby, and we couldn’t let her. So, and it’s, like, these two babies crying because they want to play together and we’re like, we can’t do that… (S125)

Another consequence of the distancing guidelines was a loss of group counselling and meetings that staff and women felt were necessary to remain connected to shelter staff and other residents. As one staff member (S118) said,

We [used to do] twice weekly psychoeducational groups… self-care and… [the] cycle of violence or something applicable to what the current kind of struggles were in the house… We had women asking for group [sessions] and we weren’t allowed to do it, and it was the piece that I missed, for sure.

Women also missed connecting with other women in shelter due to the loss of these group sessions but also pandemic protocols that encouraged distancing and separation of women and children from different families. However, women used workarounds to continue to try and connect with other residents informally, as they felt that these connections were important for fostering community and healing within the shelter: “I really bonded with all the girls… I would have loved to just sit there and support each other a bit more. We did that in our own time outside in a very informal way” (W127).

#### Restrictions on communal spaces

Communal spaces, like kitchens, TV rooms, laundry rooms, and outdoor spaces such as playgrounds, are the sites of important informal support for staff and clients, and among shelter residents. An earlier paper from our analysis clearly outlined losses of different types of space (e.g., bedrooms, bathrooms, communal spaces) in women’s shelters due to pandemic protocols [21]; here we extend this analysis to explore the impact on the quality of care and the loss of crucial connections in these spaces. Overall, shelter staff discussed uneasiness about communal spaces that were once full being empty, and the impact this had on women and children. One staff member (S105) felt that restrictions on communal spaces meant difficulties in creating a sense of community within the shelter,

[W]hat I think was a big thing, which impacted the women in shelter, is the lack of community in the shelter, because they can’t go to the communal spaces anymore… So, if [women in shelter] don’t know anyone, they could know each other, but they’re kind of not allowed to… they’re not allowed to congregate in communal spaces.

Women also reflected on activities and bonding that could be done pre-pandemic, and lamented this loss, as one woman (W124) said, “… pre-COVID you could cook for each other. Pre-COVID you could have, like, movie nights and all that kind of stuff, like get together as women…”.

Both the kitchen and dining room spaces were discussed by EDs, staff, and women as particularly difficult to lose when it came to missing the socialization among support staff, counselling staff, and women. Due to public health guidelines, many shelters shifted away from shared cooking responsibilities and congregate dining to prepared dishes delivered to women’s rooms:

It’s so weird every time I walk past the dining room now that there’s no one here, because normally this is full of people and they’re all chatting. And it’s people from all walks of life, there’s kids everywhere, everyone’s eating together and it’s like a really nice community environment. And [we] don’t get that at all right now. (S105)

Support staff (e.g., custodians, cooks) shared that due to restrictions on congregate dining, they were less able to connect with the women and their children:

Since COVID started… we don’t get as much opportunity as we would like to be able to communicate with the residents. But, we still have… our times when we go and drop food off at the door and stuff to have a little chat and check-in and see how they’re doing. They have a lot more opportunity, though, with the counsellors to be able to phone down… It’s just us as support staff, we don’t get that contact as much right now. (S104)

Even when kitchens and dining rooms were still accessible to women, rigid rules like individual cooking times and seating charts or assigned tables in the dining room impacted the community feel that used to exist. The loss of kitchen space also meant restrictions on women’s ability to cook their own meals or retrieve food items, which was no longer allowed in some agencies, as one staff member (S118) shared “… somebody couldn’t go and get themselves a glass of juice. It just felt like I was taking so much away from people who had so much taken away.” Overall, this recognition of the past success of shelter/communal spaces in terms of community engagement juxtaposed with the new realities of the pandemic created a tension for EDs, staff, and women in balancing the benefits of maintaining spaces that fostered community with practicalities of public health guidelines (e.g., sanitization, physical distancing) and the risk of viral spread.

Staff shared that changes to their shelter spaces limited the comfort that could be provided to women:

[Our shelter is] like a home. Like, when you get in there it’s very comfortable. And the women say it all the time, they don’t want to leave when it’s finally time for them to go. But now, with this COVID, it’s, like, people can’t get out fast enough. (S125)

In contrast, one ED (FG201) said that they were able to navigate public health guidelines related to distancing and communal spaces without instituting any restrictions and relying on clients and staff to follow the guidelines:

I haven’t put a single piece of tape on the floor or a single barrier in place. I think we found other ways to share communal areas, focus on infection prevention and control… our space has never been exactly what we need it to be, but at least I wasn’t in a hurry to make major changes to make it even more clinical. And all of this other stuff that we’re told is the safest way to maintain the support… We haven’t moved couches around; we haven’t done any of that.

#### The use of alternative spaces

Hotel rooms were used to expand service options when guidelines reduced available spaces in shelters; detailed findings on the use of alternative spaces can be found in a companion analysis [22]. As it relates to care interactions, women in hotels shared feelings of disconnection from staff and difficulties connecting by phone, especially if their children were with them as it was difficult to talk about their experiences of abuse with children present or to receive support by phone while also tending to their child(ren)’s needs. Creating positive relationships and rapport with women in hotels was challenging, for example,

One woman [at the hotel] preferred check-in by email. So, this is somebody I never met before and she’s emailing me every day and I’m emailing her... I’m thinking, how am I supposed to support this woman that I’ve never met, nor even spoke to on the phone?... How do you develop a rapport or relationship with somebody through email, right?... it just didn’t feel authentic. (S107)

Staff identified that it was particularly difficult to support women at hotels with their mental health or substance use needs, something they could provide better/more care for if women were staying in the shelter with more direct access to staff and support. This was also felt by women staying in hotel spaces, such as this woman (W128) who felt very disconnected from staff, “Because, clearly, nobody is taking care of me… they’re funding a [hotel] room, but I’m taking care of myself. And I’m not very good at it.”

#### Services and referrals: coordination, collaboration, and tensions

Within the GBV sector, coordination and collaboration among services is crucial as women plan their exit from the shelter, and from abuse and its effects. These community connections were challenged in the context of a global pandemic. There were particular frustrations with closures of other agencies, the turn to primarily remote work, or restrictions on service hours, which made supporting women and children much more difficult. For example, one staff member (S125) said,

Everything was shut down… you’d call housing, you wouldn’t get anybody. You’d call [income support agency] to try and set somebody up for financial assistance and it took forever for anybody to get back to you because everybody’s working from home.

Staff also felt that delays in the court system were putting women at risk and causing additional stress for other agencies that support survivors. One ED (FG205) shared their views of the housing system and restrictions on their services, indicating that delays in housing meant longer stays in shelters and more stress on women and GBV agencies,

[Housing agencies] were the first people out the door. You know, when the pandemic hit, they – they abandoned, you know, the whole thing. If we can run a shelter through COVID, why can’t [they] clean apartments or units and get them ready to get women out of [the shelter]?

For some agencies, there were delays in moving services online so regular mail services were relied on, which meant further delays in processing important paperwork, such as housing applications. Women also felt that the pandemic limited resources and in-person services, making support less accessible than it was pre-pandemic, as one (W128) said, “There’s no other resources. Like, to help maybe find a landlord or something that rents to people in a situation like this, you know… There’s the COVID. There’s not really anybody to talk to. It’s not the same.”

Staff and EDs were clear that successful coordination, usually sought by individual agencies, resulted in better support for clients and ensured that agencies had what they needed to provide comprehensive services during the pandemic. One ED (FG204) articulated:

My focus just has to be on the client and making sure at the end of the day that we’re there to support them or work with community partners that can help pick up those pieces that we’re not able to. Because, at the end of the day, we’re a community that [is] working together for the benefit of its members and we’re just one piece in the [puzzle]. But we need to collaborate more and especially with COVID. I’m not afraid to pick up the phone and ask for help…

Some of this collaboration involved looking at what services were available and providing this information to community partners:

I would say, across the sector, there’s been a reduction in beds, in availability of services for emergency residential sheltering. So, there’s been an increased collaboration between other community partner shelters to look at what’s available, how do we collectively support and coordinate care with women that are, you know, struggling in our community where we can’t support them with residential option, but we could help refer them and get them connected to a different shelter. (S120)

#### Doing trauma work: Limitations and stress

Staff also felt the negative impacts of the pandemic on themselves and their families; we have described the impact of the COVID-19 pandemic and associated guidelines on GBV staff in a related analysis [32]. However, these impacts are described here to highlight their consequences for the provision of care during the pandemic, as one staff member (S112) described, “It’s been a very different experience experiencing the same kind of trauma as my clients are experiencing at the same time and working through that.” Staff and EDs also expressed guilt and exhaustion related to the lack of referrals and services for clients, the impact that pandemic protocols had on women and children’s safety, and a focus on COVID-related tasks rather than direct work with clients. With beds in shelters greatly reduced, some staff questioned their ability to do good work or connect with clients at all: “… there were two months where I actually didn’t see any residents, because I happen to work over nights a lot… And so that also made me feel like, you know, what am I contributing, what am I doing exactly” (S103). Staff were clear that they needed to attend to their own emotional well-being to successfully provide care to clients: “… make sure that you’re taking care of your own health. Because if you’re not… how can you really be helping other people… just really make self-care a priority if it isn’t one” (S110). Opportunities to debrief and staff meetings were other suggestions provided to ensure staff wellbeing.

It is important to note that despite staff feeling like their care provision was significantly hindered by the pandemic, many women in our study expressed great understanding of the limitations of the pandemic, sympathized with the stress staff were feeling, and felt that staff were doing their best to provide support. Women were grateful that staff continued to provide services and tried to maintain a sense of normalcy in their work,

[Staff] had a lot more that they had to do every day [during the pandemic]. They worked, and when they were understaffed… it was really hard for them. So, for them to be understanding to us and be able to help us get through something, or when we go to them for something, sometimes they just had so much going on that they had [to juggle] … Because they’re people too, right, like they do their best. (W125)

### Strategies for maintaining quality of Care

#### Creative Workarounds

Shelter staff were creative in their efforts to maintain formal and informal connections and provide support to women, including alternative ways to meet (e.g., outside) and using non-traditional spaces, like parking lots, for families to see their loved ones. For example,

I’ve got one staff [member] who drives around with two lawn chairs in her trunk... and she sets out the chairs and they’re meeting wherever…. you bring them a coffee and sit in a lawn chair, you get to the depth of what’s really happening. (FG202)

Staff extended their advocacy roles to challenge rules and protocols to provide more support to women, such as allowing one family at a time to use outdoor spaces for self-care or for children to play. However, in some cases, women expressed wanting or needing more flexibility from staff and agencies when it came to balancing mental health needs and public health guidelines, as one woman (W122) shared,

I understand the seriousness of the situation, but all things considered, coming from such traumatizing experiences, I think a little bit more flexibility [was needed], with appreciation for sanitizing or whatever needs to happen to make these exceptions… just a bit more consideration of the [client’s] side of things.

#### Technology use

As reviewed above, a common approach to bridge the gap between pandemic protocols and providing service was the use of technology, either by phone (text and calling), email, or web-based video conferencing platforms, though for some agencies, texting and videoconferencing were already integrated into their service models. Within shelters, staff used email and phone calls to connect with women in quarantine, those in alternative spaces, and some staff used virtual platforms to have meetings with clients. When a shelter or alternative space was not available, staff tried to provide what support they could over the phone, such as additional safety planning or referrals. However, these strategies felt insufficient in the context of women and children having to remain at home with their abusers during a period of exacerbated stress and with limited options to leave the home due to lockdowns.

Some staff, EDs, and women indicated that the use of technology made the best of a bad situation and, despite difficulties with things like establishing rapport, staff were able to continue supporting women and children: “I’m thankful that I’m still able to connect with my clients even though it’s not face-to-face, it’s over the phone. I think that that’s been a good piece that it’s something rather than absolutely nothing” (S101). Some EDs and staff thought the continuation of a hybrid model of service, even after pandemic protocols were lifted, would be useful (depending on risk levels for each client and the type of service provided), as some women preferred this approach: “… [our outreach/counselling clients are] actually loving this stay at home, and just being able to access by phone… some of them have social anxiety, and some of them, transportation may be an issue…” (S109). However, others felt that these strategies limited staff’s ability to provide quality care, especially in a residential shelter setting, and some preferred face-to-face approaches: “… it’s great that we have [virtual platforms, but] it doesn’t replace the face-to-face bonding, connection, and ability to work through more challenging things” (FG205). Other challenges with virtual service models included concerns that not all women had access to a stable internet connection or an electronic device, safety risks (e.g., device security, children or abusers overhearing conversations, etc.), client’s potential anxiety related to technology use, and staff and women’s technological skills, which led to fewer women receiving support. One ED (FG204) shared their fears about how the switch to virtual services, and the perceived success of it, would alter services in the future,

When I read ministry [government funder] documents that are saying maximize virtual service, my fear is that that will become the new way of doing things and that people will believe that most people have access, and they don’t for a lot of reasons.

#### The provision of self-care and comfort items

Staff also provided items for self-care and activities to compensate for the unavailability of communal spaces and isolation guidelines that required women and children to spend so much time in their rooms:

But the young kids, you know, toddlers... they’re bored and so we have to constantly get creative and think of things that we can, you know, do to keep them entertained [while they’re in quarantine in their rooms]. So, we’re constantly also building like these… packages, with colouring supplies, self-care supplies, beauty supplies, like anything you can imagine… (S115)

In some shelters, televisions were added to the rooms to accommodate the loss of common rooms. Women shared appreciation for these efforts:

One of the child workers… would come around with a word of the day, a whole bunch of activities to follow and fun sort of things, and that was very helpful to kind of break up the day and give us something to actually look forward to… which was nice. (W122)

Meanwhile, other women felt more activities could have been provided, “… maybe just like have a little bit more of a broader, like for entertainment like I guess not everybody always watches TV, like maybe have books or puzzles, stuff to keep you occupied on your down time” (W127).

The provision of these items, and other basic needs, were supported in some cases by donations from the community, which typically did not slow down during the pandemic:

It was one of the most beautiful things I’ve been able to watch in my entire career, just seeing everybody come together like this… we all of a sudden had more volunteers than we knew what to do with, donations were coming in like crazy. Like, I know not everybody experienced the same thing, but it was just, it was so heartwarming to come together. (FG203)

The generosity of the community in providing support for these items was very important to leaders and staff in our study as it allowed for care to continue to be provided, as one ED (FG203) said,

[I’ve] realized how much the [location] community cares… I put out a [social media] message saying I need containers to pack food in and the restaurants… showed up on my door with thousands of boxes. And we need bottled water and [beverage manufacturer] pulls up with their truck and drops off three skids and it’s free and it’s like, ‘Use it.’

However, not all agencies were able to take advantage of increases in donations, as some had to close their donation programs due to a lack of staff, space, or pandemic protocols, which had a negative impact on the ability of staff to provide items and care to women: “We’re not even accepting used donations yet because we have limited space, so we’re struggling with how we are going to [re-open donations]” (S123).

## Discussion

Our research describes how the COVID-19 pandemic and corresponding precautions affected the quality of formal and informal care and connections that could be provided in women’s shelters and other services supporting GBV survivors. Furthermore, our research illuminates how the pandemic challenged the ability of women’s shelter staff to support women and their children experiencing violence and facilitate referrals to other services, which is well-documented in other research as an important function of women’s shelters [14, 17]. Research has established the importance of support and relationship-building between GBV service staff and survivors of violence, including women and their children [16, 38]. Our findings underscored that the pandemic impacted the ability of EDs, staff, women, and children to form strong and supportive bonds that are the basis of the care provided in women’s shelters.

In line with previous research [7, 11], our study found drastic changes to service provision in shelters, and other GBV services, due to pandemic guidelines, such as requirements for isolation, the transition to virtual services, and the reduction in available space and referrals. Our findings demonstrated how pandemic guidelines were often in conflict with the core values of GBV service organizations, which is consistent with recent research on isolation requirements and trauma-informed care [27]. Furthermore, research has highlighted the importance of the core value of Feminism in women’s shelters, something that our research found was challenged during the pandemic [39]. It is important to note that previous research has demonstrated challenges to underlying values in the GBV sector outside of the context of the pandemic, particularly for trauma-*and violence*-informed care (TVIC), such as structural and systemic barriers (e.g., lack of housing), or complex client needs (e.g., providing shelter to women actively using substances or in acute mental health distress while also providing a safe and calm environment for others in the shelter) [29, 40]. Nonetheless, our research highlighted how pandemic guidelines intensified challenges to care that was trauma-informed, particularly for isolation and quarantine requirements that mirrored the abusive behaviour that women and children were leaving behind. Our research also highlighted the complexity of intersectional factors, such as those related to mental health, disability, and gender, and the impact of experiencing multiple forms of oppression or vulnerability during a pandemic within the context of a women’s shelter. A companion paper from our data explores in more detail how the co-occurrence of the COVID-19 pandemic, the GBV pandemic, the opioid crisis, and systemic racism exacerbated challenges to the provision of services and the implementation of core values in practice [41].

Our research also highlighted the negative impact of pandemic protocols, specifically masking guidelines and physical distancing, in services with highly traumatized clients. We found that masking guidelines were difficult to implement when clients were experiencing trauma or had anxiety related to mask-wearing, and that social and visual cues were harder to read with masks, which aligns with findings from a non-GBV, therapeutic setting [26]. Furthermore, physical distancing guidelines reduced the number of available spots and clients in shelters (and encouraged the use of alternative spaces) and women and children in shelters were isolated to their rooms, requiring staff to use phone or virtual platforms to reach clients, which felt impersonal and impractical. We have explored the application of pandemic rules in the GBV sector elsewhere [28], however the current paper demonstrates that the quality of care interactions was greatly hindered by these rules and new processes. An important finding from our research is that a few staff felt that reduced capacity on-site in shelters meant fewer women on each counsellor’s caseload, allowing staff and women to spend more time together working through crucial issues like future goals and trauma. This finding suggests that funding for more staff at women’s shelters would be useful in dividing caseloads and allowing counsellors and their clients more available time to form connections, build rapport, and tailor support to the individual needs of each woman and child, supporting women’s paths to healing and moving on with life.

We have also detailed in companion analyses the use of alternative spaces, like hotel rooms, due to pandemic restrictions in women’s shelters [22] and how communal spaces were limited or eliminated in shelters [21]. The current analysis goes further to demonstrate how losses in these spaces challenged the quality of care that staff could provide to women and children, and hindered opportunities for connection, bonding, and support. These findings are novel in the context of the COVID-19 pandemic, but align with pre-pandemic research that has described the importance of communal spaces in women’s shelters [18, 20] and the importance of care interactions that take place in communal shelter spaces that fit the definition of ‘communal citizenship’ [25] as used in other literature on community-building in shelters [24]. Staff and leaders were also not immune to feeling the effects of the pandemic on their personal lives and own well-being, something which is explored in greater depth in another paper from this research [32].

Staff and EDs in our study used various strategies to try to maintain care quality, something that has been documented in other research with a more specific focus on technologically-mediated service [30, 31]. Overall, our findings support the growing evidence base on COVID-19 and difficulties with providing services virtually, including women and staff members’ varied skills and comfort with technology, as well as concerns related to safety, accessibility, and establishing rapport [13, 30, 31]. Our study also highlights novel findings related to specific, creative workarounds that staff used to reclaim care interactions with clients, such as meeting outside with lawn chairs, adjustments to harm reduction policies, TVs in shelter rooms, etc., which illuminates staff and EDs’ ongoing commitment to providing values-based, woman-centered care despite external COVID-19 guidelines that seemingly undermined these goals. A companion analysis from our research outlined the tenacity of leaders in the sector; as the pandemic pressed on, leaders began sharing their strategies with one another for how to best use, and share, resources to keep their work going [41]. The transition to new and creative strategies, however, required resources (e.g., funding or fundraising dollars, donations, supplies) that not all had access to, depending on their size and location, which ultimately limited the ability of some agencies to successfully adapt care to pandemic guidelines. Furthermore, variations in public health and funding ministry guidelines from region to region also meant variations for individual agencies in the creative workarounds that could be implemented.

### Limitations and future research

Much of our data collection took place during the first wave of the COVID-19 pandemic; research that extends beyond this period would reveal how impacts on care interactions with shelters continued as the pandemic, and related guidelines, evolved. Further, other research that extends beyond the context of women’s shelters, for example non-residential and sexual assault services, would be useful to understand how other types of GBV services were impacted.

### Policy & practice implications

Our methodologies, interpretive description and integrated knowledge mobilization, require a focus on knowledge-for-impact; we therefore offer the following recommendations to enhance care interactions now and during future crises that might alter the service landscape.

1. Pandemic protocols must consider the kind of work undertaken in shelters; changes must be grounded in core values, not be retraumatizing to women and children, and promote physical, cultural and emotional safety, including finding ways to allow ongoing formal and ideally in-person counseling, while also providing safe ways for women and children to interact informally with each other, and with staff [28]. Government and public health agencies need to work closely with GBV agencies to ensure that guidelines are implemented in a way that maintains quality of care delivered to women and their children.
2. As an integral part of positive shelter experiences, communal spaces need to be able to operate in at least some capacity during times of crisis, such as the COVID-19 pandemic. The findings of this study, in conjunction with a companion analysis [21], highlight the need for more research and funding for GBV shelter services to design new spaces that meet the existing and new needs of these organizations in ways that do not compromise the level of care and community they have historically provided.
3. What GBV services do for women and children at a time of high risk needs to be better understood by the public, government funders, and other health and social care providers. Help-seeking for violence, and especially emergency shelter stays, often activate a range of needs for families, including safe and affordable housing, physical and mental health supports, income stability, criminal justice and family legal supports, schooling changes or accommodations, etc., requiring enhanced system navigation and service access, while prioritizing physical and emotional safety. This role, uniquely performed by GBV services, is often unrecognized and under-valued [14, 24, 40].
4. New service models, while useful to many, must be developed and supported in ways that are accessible and equitable, including resources for sustainability (e.g., in software and hardware upgrades). For example, moving to technology-facilitated interactions when many women do not have stable internet access, or a safe place to have these conversations, means that in-person interactions, including shelter stays, will always be required.
5. Coordination and cooperation among agencies and services needs improvement, both in the context of an ongoing crisis, and for post-crisis planning. Closures in one part of the system, for example housing or income support offices, have serious impacts on the length of shelter stays.

## Conclusion

GBV is a serious public health issue and human rights violation that continues to negatively impact the health and wellbeing of women and children globally. Care provided in women’s shelters and related GBV services is a critical aspect of system services for women and children experiencing violence and can help mitigate impacts, such as long-term health consequences and economic hardship. However, care interactions were negatively impacted due to protocols implemented in response to the COVID-19 pandemic, ultimately, in most cases, leading to a reduction in the quality of care for survivors of violence. These impacts were most common in the usually-invisible work that shelter staff and women undertake to promote healing, develop new skills, and re-learn how to form positive and healthy relationships through supportive bonds between staff and women, and among women and children in shelters. This was acutely felt when shelters, adhering to strict physical distancing guidelines, closed communal areas that foster the sense of community and informal support that is characteristic of these spaces, and reduced, by limiting face-to-face counselling and group sessions, the ability of staff to engage in trauma work. Future emergency planning affecting the GBV sector must be done in consultation with GBV and related agencies to ensure that the predictable negative impacts of service changes can be mitigated and supportive care interactions can be prioritized.

## Data Availability

While we do not have Research Ethics Board approval to share raw data beyond the research team, interested readers may contact the first author for further study details or additional findings not reported herein.

## List of abbreviations

ED(s): Executive director(s)
FG: Focus group
GBV: Gender-based violence
ID: Interpretive description
KMb: Knowledge mobilization
PPE: Personal protective equipment
S: Staff
TVIC: Trauma- and violence- informed care
W: Woman
